# Expression of Epithelial-Mesenchymal Transition-Related Protein Claudin‐10 in Oral Lichen Planus

**DOI:** 10.7759/cureus.80696

**Published:** 2025-03-17

**Authors:** Evangelos Parcharidis, Dimitrios Andreadis, Elizabeth Lazaridou, Athanasios Poulopoulos

**Affiliations:** 1 Department of Oral Medicine and Oral Pathology, School of Dentistry, Aristotle University of Thessaloniki, Thessaloniki, GRC; 2 Second Department of Dermatology-Venereology, School of Medicine, Aristotle University of Thessaloniki, Thessaloniki, GRC

**Keywords:** claudin-10, epithelial‐to‐mesenchymal transition, immunohistochemistry, oral disease, oral lichen planus

## Abstract

Introduction: Oral lichen planus (OLP) is a common skin disease of indeterminate etiology that can affect the oral mucosa. Epithelial-mesenchymal transition (EMT) is a critical biological event that plays an essential role in several functions, such as development, tissue repair, and stem cell dynamics, but also in cancer progression. Claudin-10, an EMT-related protein, is encoded by the CLDN10 gene in humans. In the present work, we studied the immunohistological expression of Claudin-10 in OLP compared to normal oral mucosa.

Methods: Fifty-one formalin‐fixed, paraffin‐embedded samples diagnosed as OLP from patients who did not receive any medications for the treatment of OLP until the initial biopsy and ten formalin-fixed, paraffin‐embedded samples diagnosed as comprising histologically normal oral mucosa tissue from resection margins of fibromas were immunohistochemically stained and analyzed for Claudin-10.

Results: The expression of Claudin‐10 was evaluated as significantly enhanced in OLP epithelium compared to controls (*p*<0.001). In the superficial epithelial layer, the staining was markedly higher in OLP than in the controls (*p*=0.008), and in the stroma, the staining was significantly stronger in OLP (*p*=0.027). In the intermediate epithelial layer, the staining was significantly weaker in OLP than in the controls (*p*=0.001), and in the basal layer, the staining was markedly reduced in OLP (*p*<0.001).

Conclusions: The immunohistological expression of Claudin‐10 has been described and analyzed in oral mucosal disease for the first time. Our findings indicate that the expression of Claudin‐10 is dysregulated in OLP, possibly showing an interaction between the epithelium and the underlying tissue.

## Introduction

Oral lichen planus (OLP) is a common skin disease of indeterminate etiology that possibly affects the oral mucosa. A white reticular lesion is the characteristic manifestation, which may be associated with erosive and atrophic lesions [1]. The literature indicates that OLP is a relatively common disorder, with estimates suggesting it may affect up to 2% of the general population [1]. OLP is considered an oral potentially malignant disorder [2]. However, the rate of malignant transformation remains a subject of debate, primarily due to the stringent criteria employed in its diagnosis [1].

The transformation of epithelial cells into mobile mesenchymal cells, referred to as epithelial-mesenchymal transition (EMT), signifies a pivotal process in cell differentiation [3]. This transition is marked by enhanced mobility and invasiveness, attributed to reduced cell polarity and the disintegration of intercellular adhesion. Consequently, epithelial cells demonstrate the capability of transdifferentiation, converting into motile mesenchymal cells through complex biological processes. EMT is crucial in various physiological processes, including development, tissue repair, and stem cell dynamics. However, it also has pathological implications, contributing to fibrosis and facilitating the advancement of cancer [3].

Claudins, named from the Latin claudere, which means to close, are a family of critical barrier, transmembrane proteins [4], components of the epithelial intercellular tight junctions. They are structural molecules of tight junctions that are specialized membranous structures located in epithelial, endothelial, and mesothelial cells. These junctions play a critical role in regulating the permeability and polarity of cellular layers and establishing distinct compartments within cell membranes. The inflammatory process can disrupt the integrity of tight junctions, adversely affecting the formation of the cellular barrier. Consequently, this disruption may result in heightened permeability and increased exposure of underlying tissues [5].

Indeed, Claudin-10 exists in two primary isoforms, Claudin-10a and Claudin-10b. Claudin-10a’s expression is restricted to the kidney and uterus, whereas Claudin-10b can be found in many epithelia [6].

Very few research papers have investigated the immunohistological expression of the Claudins family in oral mucosal diseases. Alterations in the expression of specific proteins of the Claudins family have been observed in oral squamous cell carcinoma (OSCC) and are associated with increased malignancy and poorer prognosis [7-9]. A few papers have reported the Claudin gene expression in OLP [10,11], but none of them researched the possible alteration in the expression of Claudin-10 in OLP.

## Materials and methods

Fifty-one formalin‐fixed, paraffin‐embedded samples diagnosed as OLP (reticular and erosive clinical types) and 10 formalin-fixed, paraffin‐embedded samples diagnosed as comprising histologically normal oral mucosa tissue from resection margins of fibromas were collected from the archives of the Department of Oral Medicine and Oral Pathology, School of Dentistry, Aristotle University of Thessaloniki, Greece. All the biopsies were performed in the Clinic of the Department of Oral Medicine and Oral Pathology, Dental School, Aristotle University of Thessaloniki, Greece. 

Tissue biopsy samples were taken from patients who provided written informed consent for management, and none of them received any medications for the OLP until the initial biopsy. The Ethics Committee of the School of Dentistry, Aristotle University of Thessaloniki, Greece approved the study plan with number/date of application: 146/11-03-2022, at the meeting with protocol number 16/06.04.2022, and we were informed on April 7, 2022.

The tissue specimens were formalin-fixed in 10% buffered formalin and subsequently paraffin-embedded. The blocks were cooled on ice. Tissue sections of 4 μm were prepared and mounted on slides. The freshly cut sections were allowed to dry for several hours and then placed at 60°C overnight. In the first section, the conventional hematoxylin and eosin staining was performed to confirm the histological diagnosis. The rest of the slides were placed in xylene twice, each time for five minutes, and after that, they were placed in graded alcohol solutions 100%, 80%, and 70% sequentially for five minutes in each one of them. After this step, the slides were left in deionized water for five minutes. The antigen retrieval was performed in a microwave oven (power 850W) for twenty minutes. A Tris-EDTA solution (EnVision FLEX Target Retrieval Solution DAKO, 10mM Tris base, 1mM EDTA, pH9, diluted in deionized water 1/50) was used in all the specimens. Endogenous peroxidase was blocked with 5% hydrogen peroxide for five minutes and the Envision Flex peroxidase blocking agent (DAKO) for 10 minutes. Incubation of the slides was performed overnight at 4°C in the presence of anti-claudin-10 (1:500, ΗPA042348, Prestige Antibodies® Powered by Atlas Antibodies), a polyclonal rabbit antibody. After washing with TBS, the sections were overlaid with the peroxidase-conjugated secondary antibody for 30 min. The unbound conjugate was removed by washing it with TBS, and it was also used for all reagent dilutions and the test washes. The bound peroxidase was visualized by reaction with DAB (diaminobenzidine) and was used as a chromogen for five minutes. Finally, Meyer’s hematoxylin was used to counterstain. Neoplastic pancreatic tissue was previously documented to be positive for Claudin‐10 [12]. Consequently, human pancreatic tissue was used as the positive control. Negative controls were performed for the primary antibody (substitution of the primary antibody with either rabbit Ig or buffer TBS), the secondary antibody (substitution of the secondary antibody with buffer TBS), and for endogenous peroxidase activity (TBS buffer was used in place of all antibody samples to show the absence of endogenous peroxidase activity).

Two individual researchers (EP, AP) evaluated immunostaining manually using a conventional light microscope. The magnification for evaluating the intensity of the immunostaining was x10, whereas the magnification for evaluating the percentage of positive cell immunostaining was x40. The intensity of the staining was rated in the epithelium's basal, intermediate, superficial, and subepithelial stroma. The degree of immunohistological staining was assessed using the following brief evaluation: 0 for negative, 1 for weak, 2 for moderate, and 3 for strong intensity. The percentage of positive cell staining was assessed according to the following evaluation: 0%, 1-25%, 26-50%, 51-75%, 76-100%. Statistical analyses were conducted utilizing IBM SPSS Statistics for Windows, Version 29 (Released 2023; IBM Corp., Armonk, New York, United States), and the statistical significance was defined at 0.05 (*p*<0.05). The staining intensity and quantity were investigated with cross‐tabulation. The differences' statistical significance was examined using the non-parametric method Mann-Whitney U with an exact p‐value. Values of probability under 0.05 were considered to indicate statistical significance.

## Results

Claudin-10 was expressed with weak to moderate intensity in OLP samples in the superficial and intermediate epithelial layers and the basal layer. The staining in the stroma was mainly weak, with one sample being moderate and one negative in OLP samples. In controls, the intensity was negative to weak in superficial, moderate in intermediate, weak to moderate in basal, and negative to weak in stroma. The descriptive statistics, the rank-based comparison, and the Mann-Whitney U test of OLP and normal oral mucosa specimens' immunostaining are presented in Tables 1-3. 

**Table 1:** Descriptive statistics of Claudin-10 immunohistochemical expression in tissue layers OLP: Oral lichen planus; S/F: superficial layer of the epithelium; I/M: intermediate layer of the epithelium; BASAL: basal layer of the epithelium; STROMA: underlying connective tissue

Variable	N	Mean	Std. Deviation	Minimum	Maximum
S/F Score	61	51.48	49.188	0	180
I/M Score	61	112.13	41.798	40	180
BASAL Score	61	40.16	37.925	0	160
STROMA Score	61	17.38	11.387	0	50
OLP	61	0.84	0.373	0	1

**Table 2:** Rank-based comparison of Claudin-10 expression between OLP and control (normal) groups OLP: Oral lichen planus; control (normal): normal oral mucosa tissue; S/F: superficial layer of the epithelium; I/M: intermediate layer of the epithelium; BASAL: basal layer of the epithelium; STROMA: underlying connective tissue

Variable	Group	N	Mean Rank	Sum of Ranks
S/F Score	Normal	10	17.45	174.5
	OLP	51	33.66	1716.5
I/M Score	Normal	10	47.3	473
	OLP	51	27.8	1418
BASAL Score	Normal	10	55	550
	OLP	51	26.29	1341
STROMA Score	Normal	10	20.3	203
	OLP	51	33.1	1688

**Table 3:** Non-parametric group comparison using the Mann-Whitney U test OLP: Oral lichen planus; S/F: superficial layer of the epithelium; I/M: intermediate layer of the epithelium; BASAL: basal layer of the epithelium; STROMA: underlying connective tissue

Test Statistics				
	S/F Score	I/M Score	BASAL Score	STROMA Score
Mann-Whitney U	119.500	92.000	15.000	148.000
Wilcoxon W	174.500	1418.000	1341.000	203.000
Z	-2.671	-3.208	-4.750	-2.219
Asymp. Sig. (2-tailed) (p)	p = 0.008*	p = 0.001*	p < 0.001*	p = 0.027*
Grouping Variable: OLP. Statistical significance was set at p < 0.05. Significant results are indicated in bold with an asterisk (*).				

The key statistical measures (mean, standard deviation, minimum, and maximum) are summarized for different scores in the dataset in Table 1 (Descriptive Statistics). The dataset includes measurements of Claudin-10 expression in different tissue layers: In the superficial layer of epithelium (S/F), the mean was 51.48, with a large standard deviation (49.188), in the intermediate layer of epithelium (I/M), the mean was 112.13, the highest among layers, with a standard deviation 41.798, in the basal layer of the epithelium (BASAL), the mean was 40.16, with a standard deviation of 37.925, and in underlying connective tissue (STROMA), the mean was 17.38, the lowest layer expression, with a standard deviation of 11.387. The grouping variable is the OLP: binary variable (0=control, 1=OLP), comparing normal and OLP-affected tissues. 

The dataset in Table 2 (rank-based comparison of Claudin-10 expression between OLP and control (normal) Groups) is divided into two groups: normal (N=10) and OLP (N=51). The rank-based comparison shows each group's mean rank and sum of ranks across different scores. In the superficial layer of epithelium (S/F) score, the normal group has a lower mean rank (17.45) compared to the OLP group (33.66). In the intermediate layer of epithelium (I/M) score, the normal group exhibits a higher mean rank (47.30) compared to the OLP group (27.80). In the basal layer of the epithelium (BASAL) score, the normal group has a higher mean rank (55.00) than the OLP group (26.29). In the underlying connective tissue (STROMA) score, the normal group has a lower mean rank (20.30) compared to the OLP group (33.10). These rankings indicate significant differences between the Normal and OLP groups across all four variables (S/F, I/M, BASAL, and STROMA). The OLP group's sum of ranks is consistently higher, reflecting its larger sample size (OLP N=51 and normal N=10). 

The Mann-Whitney U test (Table 3, a non-parametric group comparison) assesses whether there is a statistically significant difference between the two groups (normal vs. OLP) for each score. This non-parametric alternative to the independent t-test is suitable for comparing two independent groups when data do not follow a normal distribution. In the superficial layer of the epithelium (S/F), the Mann-Whitney U score is 119.500, with Z = -2.671 and p = 0.008. In the intermediate layer of the epithelium (I/M), the Mann-Whitney U score is 92.000, with Z = -3.208 and p = 0.001. In the basal layer of the epithelium (BASAL), the Mann-Whitney U score is 15.000, with Z = -4.750 and p < 0.001. In the underlying connective tissue (STROMA), the Mann-Whitney U score is 148.000, with Z = -2.219 and p = 0.027. Values of p < 0.05 were considered statistically significant. Accordingly, all four scores (S/F, I/M, BASAL, and STROMA) indicate statistically significant differences. Among these, the basal layer of the epithelium (BASAL) score is the most significant, with p < 0.001, indicating the strongest statistical difference between the OLP and the normal groups.

**Figure 1:**
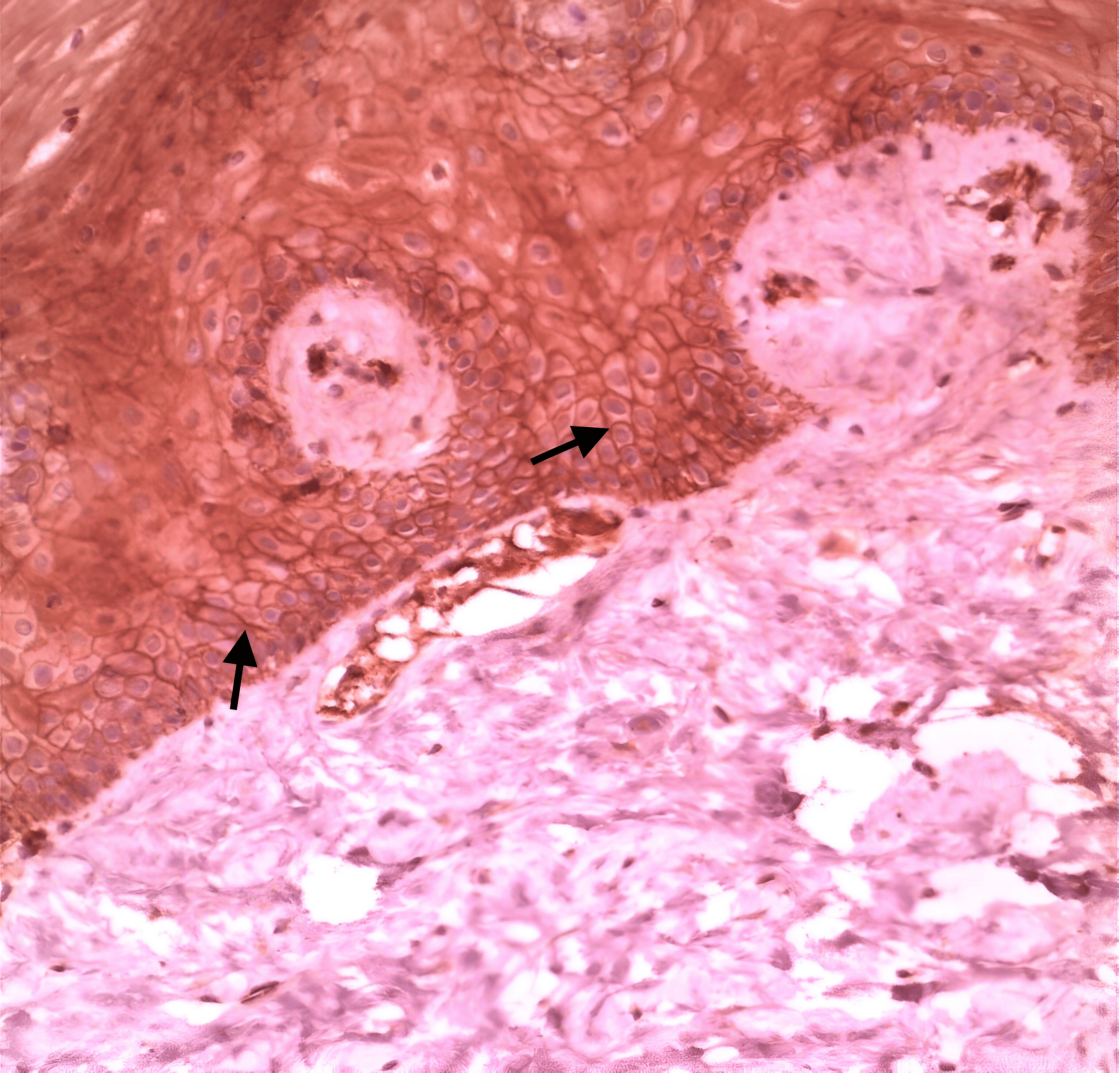
Immunostaining of Claudin-10 in normal oral mucosa with positive expression on the basal cell membranes (arrows) (original magnification x20). OLP: Oral lichen planus

**Figure 2:**
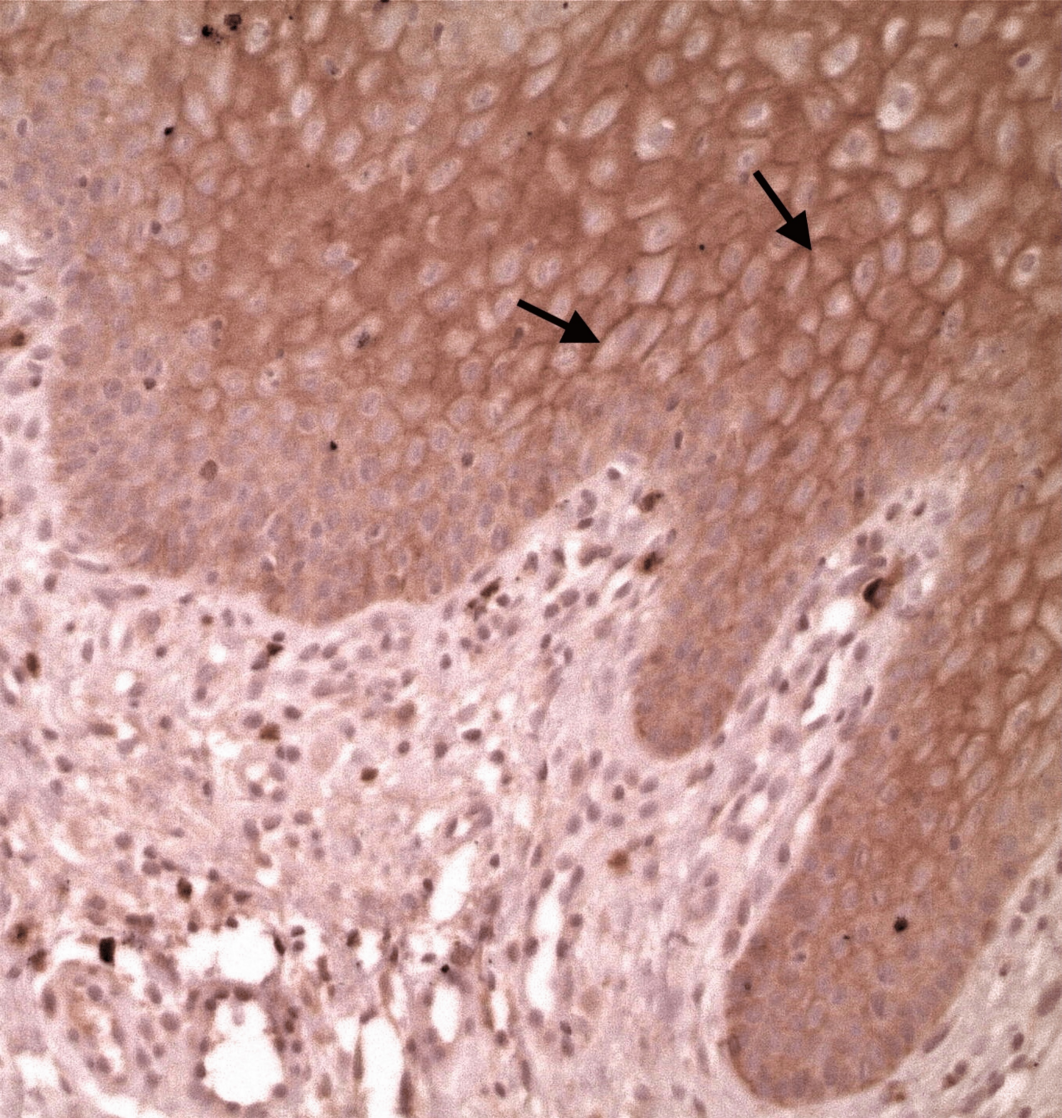
Immunostaining of Claudin-10 in OLP with positive expression on the intermediate cell membranes (arrows) (original magnification x20). OLP: Oral lichen planus

**Figure 3:**
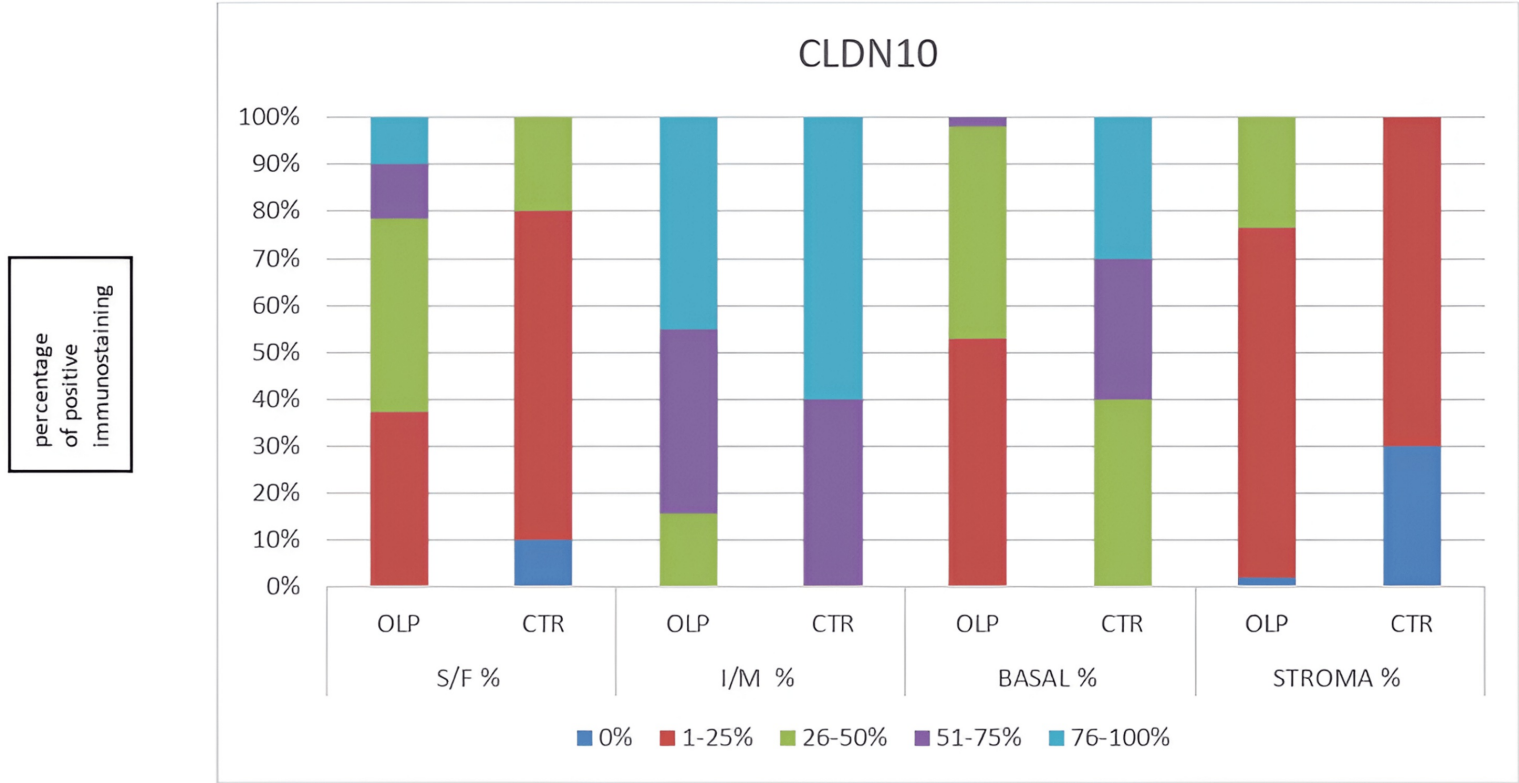
Flowchart for quantitative evaluation of the immunohistochemical staining for Claudin-10. CLDN10: Claudin-10; OLP: oral lichen planus; CTR: control; S/F: superficial layer of the epithelium; I/M: intermediate layer of the epithelium; BASAL: basal layer of the epithelium; STROMA: underlying connective tissue; % percentage of Claudin-10 positive immunostaining.

**Figure 4:**
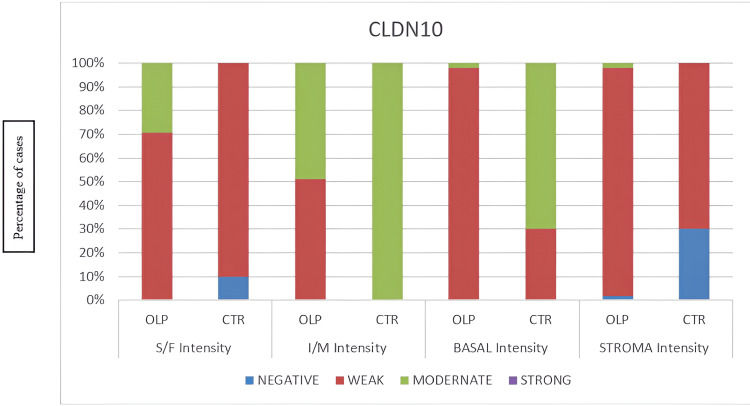
Flowchart of immunohistochemical intensity staining for Claudin-10. CLDN10: Claudin-10; OLP: oral lichen planus; CTR: control; S/F: superficial layer of the epithelium, I/M intermediate layer of the epithelium, BASAL basal layer of the epithelium, STROMA underlying connective tissue, % percentage of Claudin-10 positive immunostaining.

In the superficial epithelial layer, the immunostaining was recorded as significantly stronger in OLP than in the controls, as well as in the stroma; the immunostaining was evaluated as significantly stronger in OLP (Figure 1: normal oral mucosa specimen, Figure 2: OLP specimen). In the intermediate epithelial layer, the staining was significantly weaker in OLP than in the controls, as well as in the basal layer, the immunostaining was assessed as weaker in OLP (Figure 1: normal oral mucosa specimen, Figure 2: OLP specimen). The quantity and intensity of immunohistochemical staining are shown in the charts in Figures 3, 4, respectively.

## Discussion

This study evaluated the immunohistological expression of the EMT-related marker Claudin-10 in OLP. To date, immunohistochemistry evaluation with Claudin-10 has not been well reported in OLP. Several other Claudin family proteins, such as Claudin-1, 4, and 7, have been immunohistologically investigated [10,11].

The expression of Claudin-1, -4 [11], as well as the expression of Claudin-1, -4, -7 [10], is dysregulated in OLP, and the expression of Claudin-1, -2, -3, -4, -5, and -7 is dysregulated in OSCC [7]. Different Claudins are often dysregulated in various cancers, with their overexpression or underexpression influencing biological processes like cell proliferation, growth, metabolism, metastasis, and stemness, depending on the tumor’s origin [13].

Claudin-10 is a member of the Claudin family, which influences the progression of thyroid cancer, lung adenocarcinoma, hepatocellular carcinoma, breast cancer, ependymomas, and esophageal squamous cell carcinomas. It has been reported that Claudin-10’s decreased expression can be used as a predictor, showing a lower overall survival rate in patients with ovarian cancer [14], and its increased expression is associated with better relapse-free survival in patients with breast cancer [15].

Furthermore, Claudin-10 mutations can cause dysfunction in several tissues, including epithelia, as well as abnormalities in homeostasis of the glands, ion transport, and epidermal integrity. It is well documented that biallelic mutations in Claudin-10 cause HELIX, an acronym for the manifestations of Hypohidrosis, Electrolyte imbalance, lacrimal gland dysfunction, ichthyosis, and Xerostomia. This syndrome affects the oral cavity by the decreased secretion of saliva [16].

Our study indicates a dysregulation in the expression of Claudin-10 in OLP compared to the physiological oral mucosa. The OLP staining is significantly stronger in the superficial epithelial layer and stroma but significantly weaker in the basal and intermediate epithelial layers. These results suggest a dysregulation in cellular adhesion and polarity in OLP lesions, possibly showing an interaction between the epithelium and the underlying tissue, enhancing the potential role of EMT in the development of a distinct biological behavior of the clinical types of OLP.

The study's limitations include a small sample size and the absence of longitudinal evaluations for the patients, as OLP cases necessitate long-term follow-up, along with a lack of the Claudin-10 assessment to all the clinical types of OLP characterized by clinical diversity.

## Conclusions

To date, our research describes the immunohistological expression of Claudin‐10 for the first time in OLP lesions. The findings indicate that the expression of Claudin‐10 is clearly dysregulated in OLP, possibly showing an interaction between the epithelium and the underlying tissue. This enhances the potential role of EMT in developing a distinct biological behavior of the clinical types of OLP. There is a diversity of clinical types of OLP with distinct behavior including relapses, demonstrating the necessity of regular follow up of the OLP patients. Further research studies are needed to establish the possible association of Claudins with epithelial dysplasia in OLP and to investigate the progression of lesions potentially influenced by this characteristic.
